# Management of invading pathogens should be informed by epidemiology rather than administrative boundaries

**DOI:** 10.1016/j.ecolmodel.2015.12.014

**Published:** 2016-03-24

**Authors:** Robin N. Thompson, Richard C. Cobb, Christopher A. Gilligan, Nik J. Cunniffe

**Affiliations:** a Department of Plant Sciences, University of Cambridge, Downing Street, Cambridge CB2 3EA, UK; b UC Davis Department of Plant Pathology, One Shields Avenue, Davis, CA 95616, USA

**Keywords:** Mathematical modeling, Plant disease management, Economics of disease control, Plant trade quarantine, *Phytophthora ramorum*

## Abstract

Plant and animal disease outbreaks have significant ecological and economic impacts. The spatial extent of control is often informed solely by administrative geography – for example, quarantine of an entire county or state once an invading disease is detected – with little regard for pathogen epidemiology. We present a stochastic model for the spread of a plant pathogen that couples spread in the natural environment and transmission via the nursery trade, and use it to illustrate that control deployed according to administrative boundaries is almost always sub-optimal. We use sudden oak death (caused by *Phytophthora ramorum*) in mixed forests in California as motivation for our study, since the decision as to whether or not to deploy plant trade quarantine is currently undertaken on a county-by-county basis for that system. However, our key conclusion is applicable more generally: basing management of any disease entirely upon administrative borders does not balance the cost of control with the possible economic and ecological costs of further spread in the optimal fashion.

## Highlights

• Control of disease should balance the cost of management against its efficacy.
• Uniform control throughout administrative regions is almost always sub-optimal.
• Control in high-risk areas, informed by epidemiology and economics, is most efficient.

## 1 Introduction

The significant environmental damages and economic costs associated with invading plant and animal pathogens emphasize the importance of effective management (Pimentel et al., 2005). Epidemiological theory demonstrates how efficient control of disease requires matching the temporal and spatial scales of treatments with those of epidemics (Gilligan, 2008, Cunniffe et al., 2015a). Recent work also shows how epidemiological models can be linked to economics to balance the benefits of control against its costs (Ndeffo Mbah and Gilligan, 2010, Cunniffe et al., 2014). However, in practice, management of invading pathogens is often dominated by the geography of administrative or political boundaries. Plant trade quarantines are a notable example. For numerous plant pests and pathogens, including the emerald ash borer (Poland and McCullough, 2006), citrus greening (Stokstad, 2012) and the pine shoot beetle (Haack and Poland, 2001), initial detection triggers legislation leading to quarantine that is then applied uniformly across an entire administrative region, such as a county or state. However, pests and pathogens do not respect administrative boundaries. Therefore, control informed solely by these borders is likely to be sub-optimal.

**Fig. 1:**
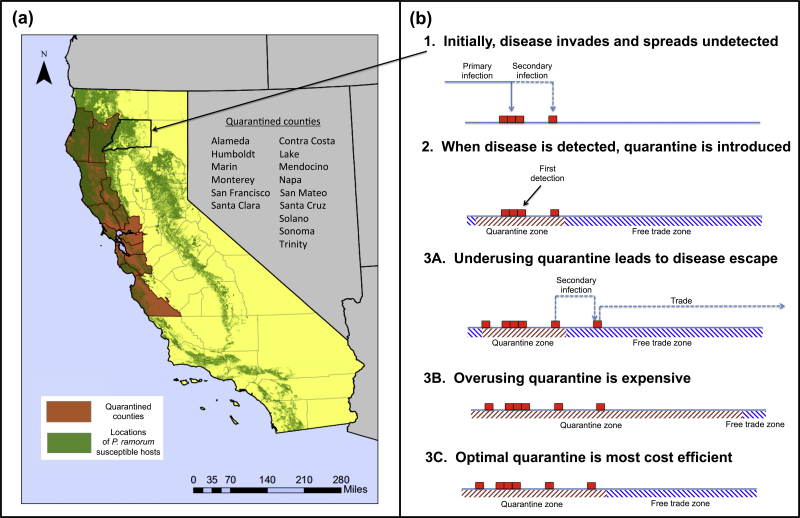
(a) Location of *P. ramorum* hosts (Meentemeyer et al., 2011) and current quarantine counties in California; (b) a schematic showing how partial quarantine could be deployed if the pathogen appears in a new county. This indicates the consequences of sub-optimal and super-optimal sizes of the quarantine region.

We illustrate the general principle using the oomycete plant pathogen, *Phytophthora ramorum*, in California as a motivating example. Sudden oak death, caused by *P. ramorum*, has killed millions of oak and tanoak across coastal California. A large number of tree and shrub species are susceptible, including many species traded by nurseries (Rizzo et al., 2005). Outbreaks in the wider environment can therefore infect nursery plants, posing risks of subsequent long-range transmission via trade (Liebhold et al., 2012). If *P. ramorum* infection is confirmed within a Californian county, legislation mandates that all nurseries across that entire county must be quarantined (APHIS, 2012). Quarantine was extended to include Trinity County in April 2015 (Fig. 1a), after the pathogen was confirmed in the county near the Humboldt County border.

As proof of concept, in the following analysis we use mathematical modeling to illustrate principles underlying an epidemiologically-informed control strategy that is potentially more cost-effective (Fig. 1b). We consider a model for an invading plant pathogen that tracks the density of susceptible and infected hosts as an initially uninfected county becomes infected. After a delay to allow first detection of the pathogen in the county, we consider the effects of applying quarantine to the plant trade in part of the county, centered on the detected outbreak. We use the model to show that quarantine extending to the borders with the neighboring counties – but not beyond – is almost always sub-optimal.

## 2 Methods

### 2.1 Mathematical model

**Fig. 2:**
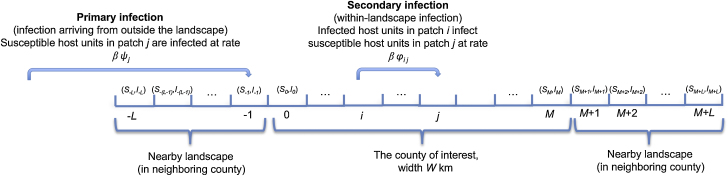
Schematic of the stochastic metapopulation disease spread model. The host landscape consists of the county where quarantine policy is being introduced (*M* + 1 patches), and nearby landscape (*L* patches on either side of the county). The pathogen spreads in the landscape via both primary and secondary infection.

Primary infection and subsequent secondary spread of a plant pathogen are modeled in a county represented by a one-dimensional landscape. The county is initially uninfected. We use a stochastic Susceptible-Infected model (Keeling and Rohani, 2008), splitting the county of length *W* into *M* + 1 equally sized patches (Fig. 2; Table S1). To allow for local bulk-up of the pathogen within each patch, we model each patch as containing a number of “host units”, corresponding to vegetation susceptible to infection. We assume that a small proportion *ρ* of hosts are in nurseries, and 1 − *ρ* are in the wider environment. The number of host units in patch *i* is *N*_*i*_ = *S*_*i*_ + *I*_*i*_, which, for each simulation, we draw from a uniform distribution (Table S1).

Motivated in the context of sudden oak death by any of the number of inland Californian counties that contain hosts susceptible to *P. ramorum* (Fig. 1), we assume that there is an inoculum source outside the county corresponding to the ongoing epidemic in the coastal region of California. After first arrival in the county, the pathogen spreads within the county via secondary infection, and there can also be additional primary infection. Since the pathogen might arrive in the county via primary infection close by, followed by secondary infection into the county, we also include *L* patches on either side of the county in our model. This is how we define our host landscape: namely, the county plus small regions on either side of the county that lie in neighboring counties.

We account for two types of spread (Grünwald et al., 2012), using a mixture of two Cauchy dispersal kernels with a short-range and a long-range component, as used in the more detailed model of *P. ramorum* by Meentemeyer et al. (2011). Secondary infection of each susceptible host unit in patch *j* by each infected host unit in patch *i* therefore occurs at average rate *βφ*_*ij*_, where *β* is the rate of infection, and where

$$\varphi_{ij}=\frac{\gamma }{N_{\text{max}}}\int_{x_{1}}^{x_{2}}{\frac{\left(1+{\left(x/\alpha_{1}\right)}^{2}\right)}{F_{1}}}^{-1}\text{d}x+\frac{1-\gamma }{N_{\text{max}}}\int_{x_{1}}^{x_{2}}{\frac{\left(1+{\left(x/\alpha_{2}\right)}^{2}\right)}{F_{2}}}^{-1}\text{d}x\text{.}$$

The integrals are over the width of patch *j*, so that *x*_1_ is the distance between the center of patch *i* and the nearest edge of patch *j*, and *x*_2_ is the distance between the center of patch *i* and the farthest edge of patch *j*. The parameter *F*_1_ is a normalizing constant so that the dispersal kernel is a valid probability distribution,

$$\int_{-\infty }^{\infty }{\frac{\left(1+{\left(x/\alpha_{1}\right)}^{2}\right)}{F_{1}}}^{-1}\text{d}x=1\text{.}$$

with a similar expression for *F*_2_. The factor of 1/*N*_max_ is the probability that a unit of inoculum lands on the particular susceptible host unit when landing in patch *j*.

Primary infection from outside the county is assumed to occur according to the same dispersal kernel as within-county spread, but assuming that there is a source of infection corresponding to *Z* infected host units a distance *d* to the west of the county. For simplicity we assume the inoculum source corresponding to this distant epidemic is of a constant size. Consequently, primary infection of each susceptible host unit in patch *j* occurs at constant per capita rate *βZψ*_*j*_, where

$$\psi_{j}=\frac{\gamma }{N_{\text{max}}}\int_{x_{1}}^{x_{2}}{\frac{\left(1+{\left(x/\alpha_{1}\right)}^{2}\right)}{F_{1}}}^{-1}\text{d}x+\frac{1-\gamma }{N_{\text{max}}}\int_{x_{1}}^{x_{2}}{\frac{\left(1+{\left(x/\alpha_{2}\right)}^{2}\right)}{F_{2}}}^{-1}\text{d}x\text{,}$$

where *x*_1_ and *x*_2_ are the distances between the primary inoculum source and the left and right edges of patch *j*, respectively.

### 2.2 Quarantine and its cost

Quarantine begins when disease is first detected in the county. Disease is detected on each host unit after a period of time beginning when the host unit becomes infected, and this period is drawn from an exponential distribution. The average period until detection for nursery hosts is assumed to be *τ*_*n*_ and for hosts in the natural environment is *τ*_*w*_.

Quarantine of nurseries occurs in the area of the county where infection is first detected, and is implemented in the obvious spatially-defined manner. For example, if *p* = 0.5 (i.e. 50% of the county is to be quarantined), and the initial detection occurs midway through the county, then the quarantine region will extend between [25%, 75%]. If instead the initial detection is 10% of the linear distance across the county, but it is still the case that *p* = 0.5, then the quarantine region is [0%, 50%].

We focus entirely on the cost of quarantine to the legislature, and consider the expected cost of deploying quarantine (*C*) over a fixed timescale *T*, after which the quarantine policy is reassessed. We assume that the cost is made up of two components: the cost of deploying quarantine, and the cost if quarantine fails,

(1) $$\begin{array}{ll} C & =C_{1}+C_{2},\\ \; & =\text{Costs associated with deploying quarantine}+\text{Expected cost due to possible trade of infected host units out of the county.} \end{array}$$

The main cost to the legislature associated with quarantine of *P. ramorum* in California is the inspection of nurseries. We therefore assume that the cost of applying quarantine is proportional to the number of host units that are affected, and denote by *η* the cost of deploying quarantine per host unit per year over the timescale *T* months. Since we assume that a fraction *ρ* of host units are in nurseries, the first term of (1) is given by

$$C_{1}=\sum_{i\in \sigma_{q}}\frac{\eta }{12}T\rho N_{i}\text{,}$$

where *σ*_*q*_ is the set of all patches in the quarantine region, so that the sum is over all patches *i* in the quarantine region.

The second term of Eq. (1), namely the expected cost due to infection being traded out of the county, is

$$C_{2}=\text{Cost if infection is traded out of county}\times \text{Prob}(\text{infection traded out of county})\text{.}$$

The first term of *C*_2_ accounts for the cost of further quarantine to the legislature after the pathogen is traded out of the county. Given the destructiveness of plant pathogens, as evidenced by large economic impacts ascribed to *P. ramorum* (Kovacs et al., 2011), we assume that this cost is a very large value, *A*. The second term of *C*_2_ can be estimated using simulations of the model. In particular, in a single simulation, if nursery host units are traded into and out of the county from uninfected areas according to a Poisson process with rate *λ*, the average proportion of host units in nurseries is *ρ*, and the probability that an infected host unit is traded without the pathogen being detected in the trade network is *q*, then the probability that a host unit that is infected for time *t*_inf_ months is transported out of the county is

$$1-\text{exp}(-\lambda q\rho t_{\text{inf}})\text{.}$$

Consequently, the probability that infection is transported out of anywhere in the entire county in this single simulation over timescale *T* months is

$$1-\text{exp}(-\lambda q\rho \sum_{j\in \Omega_{\text{inf}}}\left(T-t_{j}\right))\text{,}$$

where *t*_*j*_ is the time at which host unit *j* is infected, and where the sum is taken over all infected host units in unquarantined regions, Ω_inf_. In doing this, we assume for simplicity that quarantine works perfectly, so that infection is never traded out of quarantined areas. By averaging this expression over many simulations, we obtain the final expression for expected cost of quarantine in the county, which is

$$C=\sum_{j\in \sigma_{q}}\frac{\eta }{12}T\rho N_{i}+A\times \text{E}(1-\text{exp}(-\lambda q\rho \sum_{j\in \Omega_{\text{inf}}}\left(T-t_{j}\right)))\text{,}$$

where **E**(.) represents the averaging of this expression over many simulations of the model.

## 3 Results and conclusions

**Fig. 3:**
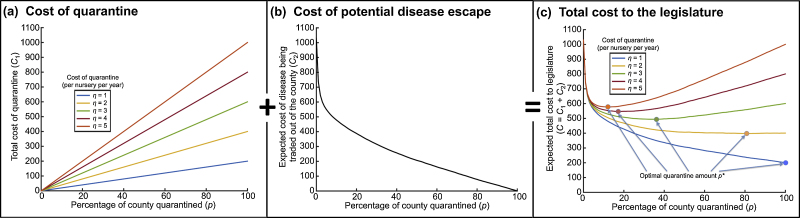
Costs to the legislature after quarantine is implemented, as the percentage of the county quarantined (*p*) varies, for different costs of implementation of quarantine (*η* per nursery per year in quarantine region): (a) cost of quarantine deployment; (b) cost of potential disease escape; (c) total cost to the legislature, evaluated as the sum of (a) + (b). Generated using 10,000 simulations of the model with the default parameter values (Table S1).

We use the model to demonstrate, by maximizing the expected cost to the legislature over a fixed time horizon, that a quarantine extending exactly to the edge of the county but no further must be less efficient than an epidemiology-driven choice of quarantine region. There is a trade-off between the cost of deploying quarantine, which increases linearly with the proportion of the county quarantined, *p*, and the chance (and therefore expected cost) of the pathogen being traded out of the region, which decreases with *p*. The optimal percentage of the county to quarantine, p*, depends on the quarantine implementation cost (*η* per nursery per year in the quarantine region); depending on *η*, it can be best to quarantine the entire county (and beyond), or control only in the vicinity of detected infection (Fig. 3). The optimal response also depends on epidemiological parameters: if the infectiousness of infected hosts is increased, then p* increases (Fig. S1a). Similarly, the more dispersive the pathogen, the greater the value of p* (Fig. S1b).

We reiterate this is a purely illustrative analysis of our principal argument, that deploying disease control measures exactly according to administrative boundaries is non-optimal in terms of cost effectiveness of control relative to risk of further spread by trade. Practical implementation of alternative interventions – for *P. ramorum* or any other pathogen – would require careful consideration and adjustment for detail. The cost function should include, for example, costs to the nursery trade, the possibility of imperfect quarantine, and careful analysis of the costs of wider-environment infection (Kovacs et al., 2011), as well as ecological costs such as damages to ecosystem services (Boyd et al., 2013). Real-world use also requires a more realistic model of host distribution and spread (Harwood et al., 2009), and might allow for temporal changes in quarantine strategies. Nevertheless, we show how the principle of linking economics and epidemiology leads to more informed control. Mathematical modeling is a key tool for this, and arguably underutilized (Cunniffe et al., 2015b). For an example of recent promising developments involving using models to inform plant disease control policy in the United Kingdom, see the UK government's Tree Health Management Plan (Defra, 2014).

Although we use sudden oak death in California as our case study, control is routinely applied in a spatially homogeneous or other relatively simple manner for both plant and animal pathogens. For example, current policy for controlling any future epidemic of Foot and Mouth Disease in livestock in the United Kingdom states that circular surveillance and control zones should be established around known infected cases (Defra, 2011). However, for both plant and animal pathogens, modeling studies suggest the optimal radius within which control should be deployed depends strongly on the local density of hosts (Parnell et al., 2010, Tildesley et al., 2009). Uniform deployment across the entire administrative region (i.e. the United Kingdom) is therefore unlikely to be most efficient. Another important example for which the link between the control strategy and the underlying epidemiology is arguably rather unclear is *Xylella fastidiosa*, a plant pathogen that is currently invading southern Italy and which leads to leaf scorch and plant dieback, thereby causing significant damage to olive groves (Stokstad, 2015). One of the main current control efforts is a 3-km wide buffer zone within which all host plants are being removed, intended to create a host-free barrier to prevent or at least greatly retard further spread. Although the buffer zone does not follow an administrative boundary, specification of its width and location would benefit from adjustment following further epidemiological investigation. In the cases of both Foot and Mouth Disease and *X. fastidiosa* it is likely that epidemiologically-motivated approaches to designing management, based on mathematical modeling of spread and control, would be most efficient.

In practice, the time required to accumulate sufficient understanding to allow reliable models to be developed might mean that, for newly invasive pathogens, informing management strategies by epidemiology might be impossible, at least initially. This is almost certainly the case for the emerging outbreak of *X. fastidiosa* in Italy. When a pathogen first arrives in a new environment, a control strategy informed by administrative borders will often be the easiest workable solution. However efficient control requires that the strategy is changed once the initial spread of the pathogen has been observed and epidemiological parameters can be inferred with more accuracy. For *P. ramorum*, as well as for Foot and Mouth Disease and numerous other pathogens of plants and animals, the epidemiology underlying spread is already well-characterized. In the specific case of *P. ramorum*, models calibrated to disease-spread data are already available and tested (Meentemeyer et al., 2011), allowing control strategies to be compared (Filipe et al., 2012). We therefore contend that this system is an example – in fact one of many – for which basing control solely on administrative boundaries leads to predictable inefficiencies that could be improved upon.
