# Supplementary material for: Management of invading pathogens should be informed by epidemiology rather than administrative boundaries
Source: Ecol Modell. 2016 Mar 24;324:28–32. doi: 10.1016/j.ecolmodel.2015.12.014 (PMC4767045; doi:10.1016/j.ecolmodel.2015.12.014)
Supplement: Supplementary file 1 [file mmc1.pdf]

Management of invading pathogens should be informed by epidemiology rather than administrative boundaries

Supplementary Material

Robin N. Thompson[a,*], Richard C. Cobb[b], Christopher A. Gilligan[a], Nik J. Cunniffe[a]

[a] Department of Plant Sciences, University of Cambridge, Downing Street, Cambridge CB2 3EA, UK

[b] UC Davis Department of Plant Pathology, One Shields Avenue, Davis, CA 95616, USA

*Corresponding author. Present address: Department of Zoology, The Tinbergen Building, South Parks Road, Oxford OX1 3PS, UK. E-mail address: robin.thompson@lmh.ox.ac.uk (R.N. Thompson).

Figure

Figure S1. The optimal percentage of the county to quarantine (p*) as a function of the cost of quarantine (η per host unit in the quarantine region per year) and the epidemiological parameters: (a) rate of infection (β); (b) scale of pathogen dispersal (α_2). In (a), β is incremented in steps of size 0.001 month^{-1} and η in steps of size 0.067. To reduce the computational resources required to produce these results, instead of running new simulations for each (η,β) pair, a database of 1000 simulations is produced for each β value examined. For each (η,β) pair, 500 simulations are then sampled out of the 1000 reference simulations. Subfigure (b) is created similarly, but with α_2 incremented in steps of 100 meters.

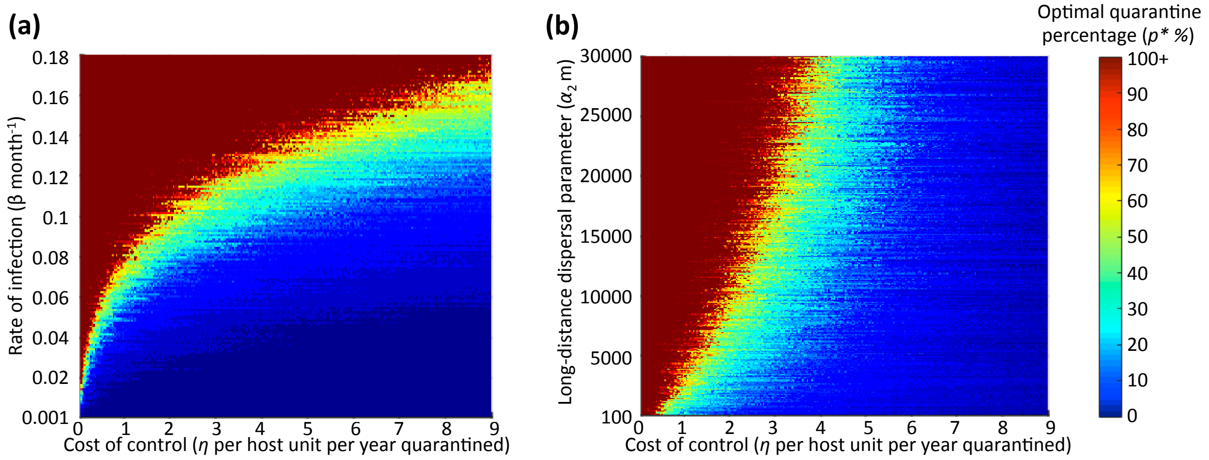

Table

Table S1. Table of parameters for the metapopulation disease spread model.

Parameter	Meaning	Value used in simulations
W	Horizontal extent of county	50 km
$M + 1$	Number of patches in the county	1000
L	Number of nearby patches considered on each side of county	200
$S_i(t)$	Number of susceptible host units in patch i at time t	Initially, all host units in the landscape are susceptible
$I_i(t)$	Number of infected host units in patch i at time t	Initially no host units in the landscape are infected
$N_i = S_i + I_i$	Number of host units in patch i	Sampled from Uniform[6,14] distribution
β	Rate of infection	0.1 month^{-1} (except where stated)
$\beta Z \psi_j$	Rate of primary infection on susceptible host units in patch j (where Z is strength of infection source outside the landscape)	$Z = 400$ host units, ψ_j given in text

$\beta\phi_{ij}$	Rate of secondary infection between infected host units in patch i and susceptible hosts in patch j	ϕ_{ij} given in text
γ	Proportion of short-range dispersal	0.99
α_1	Spatial scale of short-range spread	20 m
α_2	Spatial scale of long-range spread	10 km (except where stated)
$N_{\max}$	Maximum number of host units that any patch can accommodate	20
d	Distance between primary infection source and landscape	30 km
η	Cost of quarantine per host unit per year	See figures
T	Timescale over which quarantine is applied, which begins when the disease is first detected in the landscape	24 months
λ	Average rate at which each host unit is traded out of the county	1/12 month^{-1}
q	Probability of a traded infected host unit avoiding disease detection in the trade network	0.1
ρ	Proportion of host units in nurseries	0.01
A	Cost due to quarantine of further counties if disease escapes	10,000
τ_n	Average time after infection that disease in nursery hosts is detected	6 months
τ_w	Average time after infection that disease in hosts in the wider environment is detected	36 months
r	Average rate at which infected hosts are detected	$\frac{1}{\tau_n}\rho + \frac{1}{\tau_w}(1-\rho)$ month^{-1}
